# Illusory Body Ownership Affects the Cortical Response to Vicarious Somatosensation

**DOI:** 10.1093/cercor/bhab210

**Published:** 2021-07-08

**Authors:** Gustavo S P Pamplona, Julio A D Salgado, Philipp Staempfli, Erich Seifritz, Roger Gassert, Silvio Ionta

**Affiliations:** Sensory-Motor Lab (SeMoLa), Department of Ophthalmology-University of Lausanne, Jules Gonin Eye Hospital-Fondation Asile des Aveugles, Lausanne, Switzerland; Rehabilitation Engineering Laboratory (RELab), Department of Health Sciences and Technology, ETH Zurich, Zurich, Switzerland; Rehabilitation Engineering Laboratory (RELab), Department of Health Sciences and Technology, ETH Zurich, Zurich, Switzerland; Department of Psychiatry, Psychotherapy, and Psychosomatics, Psychiatric Hospital of the University of Zurich, Zurich, Switzerland; Department of Psychiatry, Psychotherapy, and Psychosomatics, Psychiatric Hospital of the University of Zurich, Zurich, Switzerland; Rehabilitation Engineering Laboratory (RELab), Department of Health Sciences and Technology, ETH Zurich, Zurich, Switzerland; Sensory-Motor Lab (SeMoLa), Department of Ophthalmology-University of Lausanne, Jules Gonin Eye Hospital-Fondation Asile des Aveugles, Lausanne, Switzerland; Rehabilitation Engineering Laboratory (RELab), Department of Health Sciences and Technology, ETH Zurich, Zurich, Switzerland

**Keywords:** affective, brain, fMRI, multisensory, pain, robotics, sensorimotor, touch

## Abstract

Fundamental human feelings such as body ownership (“this” body is “my” body) and vicariousness (first-person-like experience of events occurring to others) are based on multisensory integration. Behavioral links between body ownership and vicariousness have been shown, but the neural underpinnings remain largely unexplored. To fill this gap, we investigated the neural effects of altered body ownership on vicarious somatosensation. While recording functional brain imaging data, first, we altered participants’ body ownership by robotically delivering tactile stimulations (“tactile” stroking) in synchrony or not with videos of a virtual hand being brushed (“visual” stroking). Then, we manipulated vicarious somatosensation by showing videos of the virtual hand being touched by a syringe’s plunger (touch) or needle (pain). Only after the alteration of body ownership (synchronous visuo-tactile stroking) and specifically during late epochs of vicarious somatosensation, vicarious pain was associated with lower activation in premotor and anterior cingulate cortices with respect to vicarious touch. At the methodological level, the present study highlights the importance of the neural response’s temporal evolution. At the theoretical level, it shows that the higher-level (cognitive) impact of a lower-level (sensory) body-related processing (visuo-tactile) is not limited to body ownership but also extends to other psychological body-related domains, such as vicarious somatosensation.

## Introduction

A proper sense of body ownership (the feeling that “this” body is “my” body) is the essential precursor of our ability to physically interact with the environment and is based on the integration of different sensory inputs (Blanke and Metzinger 2009). A similar exploitation and combination of percepts from different senses is also at the basis of the sense of vicariousness, the ability to understand, and almost “feel” the sensations of others (Bufalari and Ionta 2013). A possible relationship between body ownership and vicariousness has been proposed on the basis of vicariousness-relevant effects of the so-called “rubber hand illusion” (RHI) (Durgin et al. 2007; Schütz-Bosbach and Prinz 2007): a well-known experimental protocol able to produce illusory changes in body ownership, inducing the feeling that a fake hand (rubber hand) belongs to oneself via a visuo-tactile multisensory conflict (Botvinick and Cohen 1998). According to the RHI protocol, participants observe a dummy hand being stroked (visual stroking), while their own hidden hand is also stroked (tactile stroking). When the visual and tactile stroking are synchronous (the same regions of the dummy and the participant’s hand are stroked at the same time), participants’ feeling that the rubber hand belongs to them is stronger than when the visuo-tactile stroking is asynchronous (Tsakiris and Haggard 2005; Aimola Davies et al. 2010).

At the behavioral level, the relationship between body ownership and vicariousness has been demonstrated based on the expression of vicarious feelings for the dummy hand (Fahey et al. 2019), different sensitivity to the RHI as a function of different responsiveness to vicariousness (Botan et al. 2018), the decrease (Paton et al. 2012; Ide and Wada 2017), or delay of the RHI (Cascio et al. 2012) in association with low vicariousness, and the over-augmentation of the RHI in vicariously hypersensitive populations (Derbyshire et al. 2013).

At the neural level, despite some indirect observations, the existence of a similar link between body ownership and vicariousness remains largely unexplored. In particular, like the RHI has been associated with the activation of a specific brain network comprising mainly the temporo-parietal junction (TPJ) (Martinaud et al. 2017; Convento et al. 2018; Wawrzyniak et al. 2018) and premotor cortex (PMC—Ehrsson et al. 2004; Olive et al. 2015; Zeller et al. 2016; Guterstam et al. 2019), also the experience of vicarious somatosensation has been linked to temporo-parietal and premotor activations (Avenanti et al. 2005; Shimada et al. 2016; Vandenbroucke et al. 2016; Bowling and Banissy 2017; Grice-Jackson et al. 2017; Benuzzi et al. 2018; Flasbeck et al. 2019; Bukowski et al. 2020; Ionta et al. 2020; Tholen et al. 2020).

Despite such overlaps, it is still unclear whether changes in body ownership are associated with neural modulations within regions encoding vicariousness. To fill this gap, in the present study, we used a within-subject design to directly compare the effects of either inducing or not changes in body ownership on the neural response associated with vicarious experience of somatosensation. To this aim, we exploited robotics compatible with magnetic resonance (MR) imaging to combine a variant of the RHI (virtual hand illusion—VHI) (Perez-Marcos et al. 2012; Pyasik et al. 2020) with functional MR imaging (fMRI) of the brain activity associated with vicarious experience of different somatosensations (pain, touch).

We hypothesized that if the neural underpinnings of body ownership and vicariousness are related, then inducing a change in body ownership should affect the activity of regions encoding vicarious somatosensation, with a particular focus on TPJ and PMC. To test this hypothesis, we induced the VHI by using the MR-compatible robot to deliver a tactile stroking to the participant’s hand, while the visual stroking was represented on a virtual human hand shown through MR-compatible goggles. Immediately after such visuo-tactile stimulation, participants underwent a protocol to induce vicarious somatosensations: observing videos of the virtual hand being touched by the plunger of a syringe (vicarious touch somatosensation) or pinpricked by the same syringe (vicarious pain somatosensation). This distinction between different types of vicarious somatosensation was used to investigate whether the influence of altered body ownership would impact vicarious somatosensation as a whole, or rather specifically for a particular type of vicarious somatosensation.

## Materials and Methods

### Participants

Fifteen healthy, right-handed, young adults (age = 24.7 ± 3.2 years-old, 8 women) participated in the study. Hand dominance was assessed through the Edinburgh inventory for handedness (Oldfield 1971). Exclusion criteria were left-handedness, vision impairments, contraindications to fMRI, and history of mental and/or cardiovascular disorders. All participants signed an informed consent form prior to the inclusion in the study. The study was approved by the local Ethics Committee and was performed in accordance with the Declaration of Helsinki (2013).

### Procedure

**Figure 1:**
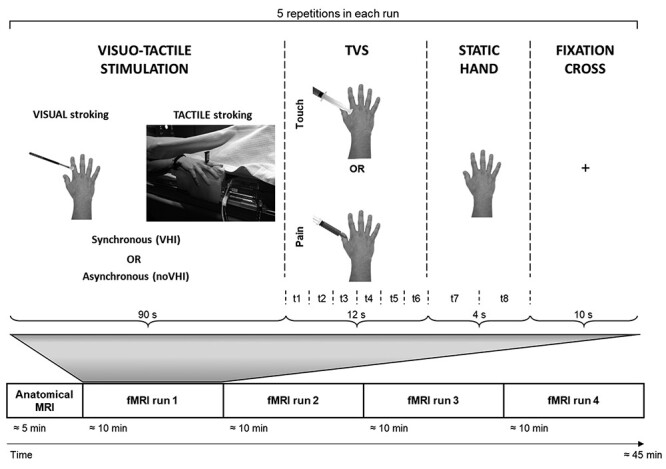
Experimental Design. Each of the four fMRI runs comprised five repetitions. Each repetition started with a block of visuo-tactile stimulation to either induce (VHI) or not (noVHI) the illusion of owning the virtual hand (90 s). This was followed by a block of different TVS, comprising videos of either vicarious touch (the virtual hand being touched by the syringe plunger) or vicarious pain (the virtual hand being pricked by the syringe needle). Each video lasted 4 s and was repeated three times in a row. The total duration of the TVS block was 12 s. Then, a picture of a static hand with the syringe absent was presented (4 s). Finally, a block of fixation cross was presented as the common baseline (10 s). Abbreviation: *t*1–*t*8 (time-bins for the time-course evaluation).

Before the experiment, participants received instructions to stay still and relaxed on the MR bed, with the eyes open during the whole experiment. Then, they were asked to lie down supine on the MR scanner bed and to place their right hand on the hand support of the robotic device. To ensure sensitivity to the visuo-tactile stimulation, during the setup, all participants were asked to confirm that they felt the brush stroking on their index finger and that they saw sharply a template image via the goggles. The experimental session comprised four runs in a single experimental session (Fig. 1). Each run included phases of synchronous or asynchronous visuo-tactile stimulation followed by the vicarious touch or pain somatosensations, the static hand observation, and the fixation cross (baseline). Such a sequence was repeated five times in each run. Two visual and two tactile stimulation profiles were predefined and presented in a counterbalanced way; the same number of synchronous and asynchronous visuo-tactile stimulations was provided. Thus, the phases of vicarious somatosensations could be preceded by either synchronous (VHI condition) or asynchronous (noVHI condition) visuo-tactile stimulation, resulting in four experimental conditions: Touch|VHI, Pain|VHI, Touch|noVHI, and Pain|noVHI, grouped in a 2 × 2 factorial design, in which the factors were visuo-tactile “Illusion” (VHI, noVHI) and “Type of Vicarious Somatosensation” (TVS; Touch, Pain). As each run comprised five blocks and we had four conditions, one of the conditions was presented twice in each run; at the end of the session, all conditions were presented five times in total, in a counterbalanced manner. The whole imaging protocol lasted about 45 min.

### Virtual Hand Illusion

**Figure 2:**
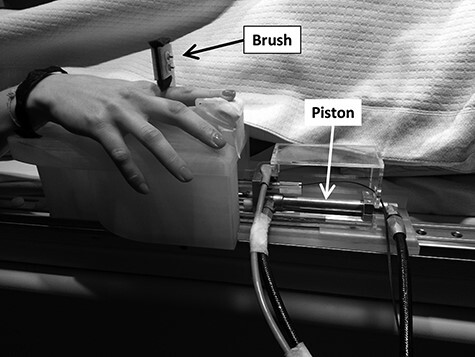
Tactile stimulation. The MR-compatible segment of the robot was attached to the MR bed and used to provide the tactile stimulation: stroking of the participants’ right index finger. The robotic movement was provided by the master part of the robot with predefined, automated trajectories, located outside the MR scanner room.

Visual stimuli consisted of videos displayed via MR-compatible goggles, placed at 3–5 cm in front of the participant’s eyes, and adjusted to achieve a clear dual-view image covering the entire visual field (Resonance Technology, Inc.). According to the VHI procedure, the videos showed a virtual right human hand being brushed by a paintbrush (Tagini et al. 2019; Pyasik et al. 2020) on the dorsum of the index finger. At the same time, synchronously or asynchronously with respect to such visual stroking, a tactile stimulation was provided to the dorsum of the participants’ right index finger through the MR-compatible robot (Gassert et al. 2006; Ionta et al. 2011) (Fig. 2). The synchrony between the visual and the tactile stroking was automated (trajectories were previously defined) and monitored (trajectories could be viewed in real-time and were then saved) according to two experimental conditions. Only in the synchronous condition, the visual and the tactile stroking were matching, thereby inducing the VHI (Riemer et al. 2019). The tactile brushing of the participant’s finger was performed by the robot following the same onset, location, direction, and speed of visual brushing of the virtual hand’s finger performed by the virtual brush. During the asynchronous pattern, the visual stroking and the tactile stroking were different in terms of onset, location, and direction. This resulted in a match or mismatch between the location on the participant’s hand, where the tactile brushing was felt and the location on the virtual hand where the visual brushing occurred.

The robot was mounted on the side of the MR scanner bed in a position adjustable according to the length of the participant’s right arm. The tactile stimulation was delivered by the slave part of the robot, attached to the MR scanner bed, and actuated by a hydraulic piston that mirrored the movement of a master piston located in the control room through a hydrostatic transmission. The slave part was designed to move a piece of foam (brush) along one degree of freedom through a linear carriage to provide the tactile stimulation. The system was controlled by a dedicated computer equipped with a data acquisition card (PCI 6221, National Instruments) and customized code written in LabVIEW (Laboratory Virtual Instrument Engineering Workbench, National Instruments).

### Vicarious Somatosensation

Immediately after the VHI procedure, during fMRI data recording participants observed other videos (Fig. 1) showing one out of two types of vicarious somatosensation: 1) for the vicarious touch, the plunger of a hypodermic syringe was approaching and touching the index finger of the virtual human hand; 2) for the vicarious pain, the same syringe was approaching the hand from the needle side and it pricking the index finger of the virtual hand in the same location where it was touched during the vicarious touch somatosensation. In both types of vicarious somatosensations, the syringe moved freely (i.e., was not held by anyone), and the video lasted 3.8 s with a 0.2-s period of white screen in-between and was repeated three times (12-s total duration). Each block of vicarious somatosensation was followed by a block of “static” condition, showing the same virtual hand without the approaching syringe (4 s). To minimize visual habituation, the inner content of the syringe could be either red or yellow, presented in a balanced way. Videos of both vicarious somatosensations were presented in a fixed pseudo-randomized sequence and counterbalanced across participants. Each participant underwent four consecutive fMRI data acquisition runs and was instructed to carefully watch the videos without any explicit request to empathize with or take the perspective of the virtual hand.

### Psychological States

**Figure 3:**
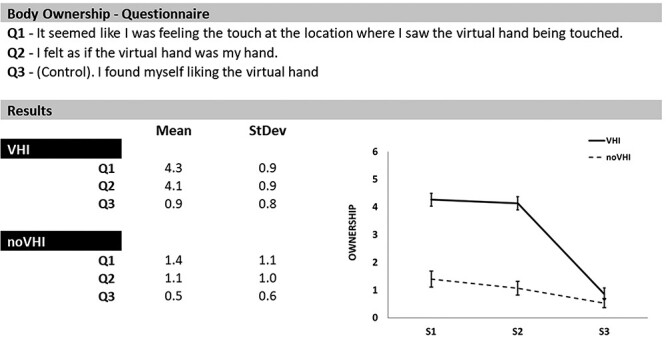
Psychological state measures. Participants scored three typical items from of the RHI questionnaire about their conscious feelings during both synchronous (VHI) and asynchronous (noVHI) visuo-tactile stroking (Top rows). Participants’ ratings for each question (Q) indicated stronger feelings of embodiment for the virtual hand during VHI than noVHI visuo-tactile stimulations (Bottom rows). Graphical representation of the participants’ ratings (Bottom-right panel). Asterisks represent significant differences. Error bars represent standard errors.

To assess individual susceptibility to alterations of body ownership due to the VHI, at the end of the experiment, participants completed a three-item questionnaire adapted from the original RHI questionnaire of Botvinick and Cohen (1998) (RHI Qs), with reference to the sensations they felt during both the synchronous and asynchronous visuo-tactile stimulation, separately (Fig. 3, top rows). The three items were presented in randomized order. The first two items of the RHI Qs (Q1, Q2) concerned illusory ownership for the virtual hand. The third item (Q3) was administered as control. To evaluate their sensations, participants completed the RHI Qs indicating their level of agreement with Qs with respect to both synchronous and asynchronous visuo-tactile stimulations. Ratings were given according to a seven-point Likert scale ranging from 0 (“I totally disagree”) to 6 (“I totally agree”) corresponding to the participant’s evaluation of the specific Q during the either the synchronous or asynchronous visuo-tactile stimulation. As an index of altered body ownership, the scores of the RHI Qs with reference to either the synchronous or asynchronous visuo-tactile stimulation were compared with each other by means of Bonferroni-corrected paired *t*-tests (*P* < 0.025) (Palluel et al. 2011).

### Psychological Traits

To evaluate participants’ emotional sensitivity/reactivity, prior to the experimental session, all participants completed the Interpersonal Reactivity Index (IRI; Davis 1983), which comprises four subscales commonly used to assess: the tendency to experience sympathy/compassion for others (Empathic Concern); how much personal distress results from witnessing others in distress (Personal Distress); the disposal to adopt others’ perspectives (Perspective Taking); and the tendency to get imaginatively involved with fictional characters/situations (Fantasy Scale). All subscales’ IRI scores have been computed according to the standard procedures (Davis 1983) and statistically analyzed and compared with each other by means of paired *t*-tests (*P* < 0.05).

### MRI Data Recording

Functional and anatomical MR imaging data were acquired with a Philips Achieva 3-Tesla MR scanner, using a 32-channel head coil (Philips). Functional runs comprised 290 functional volumes, obtained by a *T*_2_*-weighted gradient-echo planar imaging single-shot sequence (repetition time/echo time (TR/TE), 2000/30 ms; flip angle, 82°). Thirty-five contiguous axial slices with a thickness of 3.00 mm and an interslice gap of 0.7 mm were acquired in ascending order (in-plane resolution, 3 × 3 mm^2^; field of view, 240 × 240 mm^2^). Five dummy scans were acquired in order to avoid acquisition during incomplete magnetization effects period, and the total duration of each functional scan was 9min53s. High-resolution, *T*_1_-weighted, anatomical images were acquired, intended for structural reference, using the Magnetization Prepared Rapid Gradient Echo sequence and the following parameters: TR/TE, 6.77/3.10 ms; in-plane resolution, 1.03 × 1.03 mm^2^; slice thickness, 1.20 mm, field-of-view = 225.58 × 174.00 mm^2^, number of slices, 145; flip angle, 9°; total duration, 4′44″.

### MRI Data Analysis

Functional and anatomical MR imaging data preprocessing was performed with SPM12, implemented in MATLAB (R2017b, The MathWorks). Functional images were slice-time corrected by using the middle slice as the reference, and head movement was corrected by realigning all volumes for each participant to the mean image generated by each run. Then, we coregistered the anatomical image to the space of the mean functional image and performed the segmentation in the resulting anatomical image, creating masks for gray and white matter and cerebrospinal fluid compartments. During the segmentation step, a forward deformation field was also created, subsequently used to perform spatial normalization of the functional images from each run to the Montreal Neurological Institute (MNI) template. We performed spatial smoothing of the resulting functional images using a Gaussian kernel of 8-mm full width at half maximum.

The preprocessed images were analyzed through the General Linear Model (GLM), in which we modeled 1) a block design, to verify for sustained activations during the whole period of vicarious somatosensation, and 2) an event-related design based on Finite Impulse Response (FIR) basis functions, to assess transient blood-oxygen-level-dependent (BOLD) signal time-courses during the period of vicarious somatosensation and the subsequent static hand.

For the sustained activation analysis (block design—computed for the whole brain, see Sustained activation analysis), the regressors of interest were Touch|VHI, Pain|VHI, Touch|noVHI, and Pain|noVHI, specified by boxcar functions with a 12-s duration, representing the vicarious somatosensation and the participant-specific onsets to measure sustained brain activity. These boxcar functions were convolved with a hemodynamic response function (HRF), therefore, assuming a model that follows a canonical shape.

For the transient activation analysis [region-of-interest (ROI)-specific event-related approach—computed for a set of specific brain regions, see Transient activation analysis], the 12-s vicarious somatosensation plus the subsequent 4-s static-hand periods were further subdivided into eight time bins of 2 s each—the temporal resolution of the functional acquisition. This resulted in 32 regressors of interest—the same four regressors as from the block design but subdivided into time-bins wherein a predefined response shape was not assumed. Syringe color was not taken into account in the analysis. As regressors of no interest, we included, in the model, the six parameters of head movement (representing translation and rotation). In order to remove unwanted low frequencies while preserving experimental variance despite the long intervals between conditions (the vicarious somatosensation blocks), a 150-s-cutoff high-pass filter was used (higher than the SPM default of 128 s). An auto-regressive model of order 1 was employed to account for temporal autocorrelation due to unmodeled nuisance signals.

### Sustained Activation Analysis

Whole-brain analysis was performed using a block design model to verify the sustained brain activation differences between the defined conditions throughout the brain (Supplementary Fig. 1*A*). Beta values representing the regressors of interest (Touch|VHI, Pain|VHI, Touch|noVHI, and Pain|noVHI) were estimated and contrast images for each condition with respect to the baseline (fixation cross) were created for each block and each participant. Contrast images of the same condition were then averaged across the five blocks, resulting in four contrast images per participant. Thus, we created new contrast files for every participant by computing the interaction and main effect contrasts (products of a two-way repeated-measures ANOVA) using the ImCalc function in SPM12. Therefore, the main effect of the visuo-tactile stimulation (factor “Illusion”) referred to the comparison between the brain activity after VHI and noVHI, while the main effect of the TVS referred to a comparison between the brain activity associated with the observation of “Touch” and “Pain” videos.

The described approach of obtaining interaction and main effect contrasts in the first level before being taken to the second-level is recommended for SPM in the case of within-subject factorial designs (Henson 2015; McFarquhar 2019). This partitions the GLM error into separate components and offers the advantage of no concerns about nonsphericity while maintaining false-positive control (compared with pooled error). One-sample *t*-tests were carried out to identify the common activations/deactivations across participants for each particular interaction or main effect. The resulting maps were thresholded at the voxel level at *P* < 0.005 and corrected for multiple comparisons using a false discovery rate (FDR) at the cluster level at *P* < 0.05. A cluster-wise extent threshold of 10 voxels was also applied. Only for purposes of interpretation of the results, we extracted the average beta value (parameter estimate) from the activated clusters using MarsBaR (http://marsbar.sourceforge.net; Brett et al. 2002) and built boxplots for each condition separately. For significant clusters and for purposes of visualization of the effects over time, we extracted the average beta across participants for each effect and computed the pairwise effect size using Cohen’s *d* for *t*-tests. Graphs for visualization were generated using the ggplot2 library. Moreover, to support interpretation of the sustained activation results, extracted average event-related contrasts from the activated clusters were also obtained and FIR time-courses (see procedure below) for each condition were plotted. Visualization of whole-brain results was obtained via MRIcroGL software and boxplots and FIR time-courses were generated via the ggplot2 library in RStudio.

### Transient Activation Analysis

ROI analysis was performed using the event-related design, to check for transient brain activation between the defined conditions in the hypothesized ROIs at each of the eight time-bins comprised in each block. This allowed us to apply a less restrictive statistical threshold, as this approach reduces the number of multiple comparisons. These ROIs represented specific areas of the brain thought to be related to the neural mechanisms involved in the experiment (Supplementary Table 1). In order to avoid circular analysis, 18 hypothesized ROIs were defined through independent sources (Kriegeskorte et al. 2009).

A first set of ROIs was defined on the basis of previous related studies (Bingel et al. 2004; Mayka et al. 2006; Ionta et al. 2011; Flasbeck et al. 2019). The peak coordinates of these ROIs were converted to the MNI space, when necessary, using the function tal2icbm_spm (http://brainmap.org/icbm2tal/). Then, 10-mm-radius spheres (fixed number of voxels) centered in those coordinates were created using MarsBaR. According to this procedure, we defined the following ROIs: TPJ, extrastriate body area (EBA), PMC, primary sensory cortex (S1), and secondary somatosensory cortex (S2), supramarginal gyrus (SMG). Supplementary Table 1 shows the selected ROIs and their respective automated anatomical labeling labels (Tzourio-Mazoyer et al. 2002), center coordinates, and number of voxels.

A second set of ROIs was defined from meta-analytic maps, downloaded from Neurosynth (http://www.neurosynth.org; Yarkoni et al. 2011) after entering the following terms: anterior cingulate cortex (ACC), anterior insula (ant Ins), precuneus (preCun), thalamus (Thal), and supplementary motor (SMA). Then, images were thresholded using the ImCalc function in SPM12 and using different z-score values depending on the anatomical features of the region, that is, the number of voxels in these clusters varied for each ROI. Clusters of interest were isolated and saved using MarsBaR.

From each ROI, average betas of activation (parameter estimates) for the conditions Touch|VHI, Pain|VHI, Touch|VHI, and Pain|VHI minus baseline were extracted for each time bin using MarsBaR. Then, these betas were further averaged across runs for each participant. This approach enabled us to analyze the data using a three-way repeated-measures ANOVA (hereafter “All Effects” analysis) implemented in RStudio (stats library) with the factors Illusion (VHI, noVHI), TVS (Touch, Pain), and Time (eight time bins) (Supplementary Fig. 1*B*). For a complete representation of the ROI-specific results of the All Effects analysis, the three- and two-way interactions were considered significant according to a liberal statistical threshold (unc. *P*-values < 0.05), further corrected for multiple comparisons using FDR computed at the ROI level (18 ROIs) to reflect the strength of each result. Furthermore, post-hoc analyses were performed to explore the significant three- and two-way interactions across time bins (hereafter “Differential,” “Illusion-related,” and “TVS-related” analyses). In the “Differential” analysis, significant three-way interactions were further analyzed through pairwise comparisons of differences of vicarious pain minus touch (Pain-Touch) between VHI and noVHI separately for each level of the factor Time. In the “Illusion-related” analysis, significant two-way interactions Illusion × Time were further analyzed through pairwise comparisons between VHI and noVHI separately for each level of the factor Time. In the “TVS-related” analysis, significant two-way interactions TVS × Time were further analyzed through pairwise comparisons between Touch and Pain separately for each level of the factor Time. Finally, in order to assess which type of vicarious somatosensation was driving the results, for each ROI showing three- or two-way significant interactions, we further analyzed the pairwise comparisons between VHI and noVHI for Touch and Pain separately for each level of the factor Time (hereafter “TVS-specific illusion” analysis). For all analyses, post-hoc analyses were conducted with the emmeans package (Lenth et al. 2018) in R and Tukey correction for multiple comparisons across time-bins was performed. For all significant effects, the effect size was computed using partial effect-sizes (η^2^) for ANOVA tests using the DescTools library and the Cohen’s *d* for the pairwise comparisons. Graphs for visualization were generated via the ggplot2 library.

## Results

### Psychological States (RHI Qs)

In regard to body ownership, participants reported stronger feelings of illusory ownership (*Q*1, *Q*2) for the virtual hand during the synchronous than the asynchronous visuo-tactile stimulation (all *P* < 0.001). In particular, scores for *Q*1 and *Q*2 were higher for the synchronous (*Q*1 = 4.3; *Q*2 = 4.1) with respect to the asynchronous (*Q*1 = 1.4; *Q*2 = 1.1) visuo-tactile stimulation (all *P* < 0.001). The scores for the control item (*Q*3) were not statistically different between synchronous and asynchronous visuo-tactile stimulation (*P* = 0.1) (Fig. 3, bottom rows and right panel).

### Psychological Traits (IRI)

IRI scores for Personal Distress [mean = 14.6 (standard deviation = 4.1)] were the highest, followed by Fantasy Scale [11.7(4.6)], Empathic Concern [9.7(4.1)], and Perspective Taking [7.1(3.6)] (all *P*s < 0.05). These findings were in line with previous studies (Singer et al. 2004; Lamm et al. 2007; Costantini et al. 2008; Ionta et al. 2020) and suggested our sample’s general emotional sensitivity/reactivity.

### Sustained Activation

**Figure 4:**
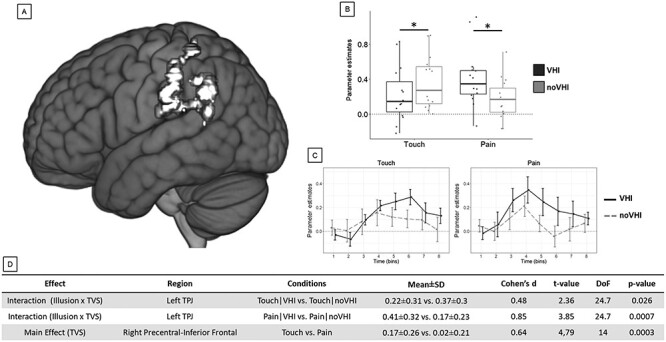
Sustained activation, interaction. (*A*) Left TPJ was the only region in which the whole-brain analysis indicated the significant interaction between the factors Illusion (VHI, noVHI) and TVS (Touch, Pain). (*B*) After synchronous visuo-tactile stroking (VHI), the sustained activity in TPJ was lower during vicarious touch and higher during vicarious pain, compared with asynchronous visuo-tactile stimulation (noVHI). Asterisks represent significant differences. (*C*) Temporal profiles extracted from the detected cluster are shown for each condition separately. Especially after the VHI visuo-tactile stimulation, the peak of activation during vicarious touch (6th time bin) was reached later than during vicarious pain (4th time bin). (*D*) Descriptive statistics of all significant effects (Effect), in the given relevant region (Region), for the specific contrast between two conditions (Conditions) and their values (Mean ± SD).

The two-way repeated-measures ANOVA performed at the whole-brain level showed the positive interaction between Illusion (VHI, noVHI) and TVS (Touch, Pain) in the left TPJ (Fig. 4*A*). Post-hoc tests of this interaction indicated that, for vicarious touch, the activation during Touch|VHI was significantly lower than during Touch|noVHI (Fig. 4*B*). The inverse was observed for vicarious pain, in which the activation during Pain|VHI was significantly higher than in Pain|noVHI (Fig. 4*B*). The descriptive statistics for all significant effects are reported in Figure 4*D*. In addition, the main effect of TVS was significant in a cluster comprising the right precentral and inferior frontal gyri, indicating higher activation during vicarious touch than during vicarious pain (Supplementary Fig. 2*A*,*B*). The main effect of Illusion (VHI, noVHI) was not statistically significant in any voxel.

### Transient Activation

By analyzing sustained activation results over Time (Fig. 4*C* and Supplementary Fig. 2*C*), we found that the significant differences found with the sustained activation approach might be spurious, because of the different temporal profile of the hemodynamic response associated with the different types of vicarious somatosensation. Especially after the VHI visuo-tactile stimulation, the BOLD signal peaked and decreased more slowly for vicarious touch than for vicarious pain. This effect might affect results obtained from analysis approaches that apply the same predefined block-related HRF model to two activation profiles (Touch, Pain) that are temporally different.

**Figure 5:**
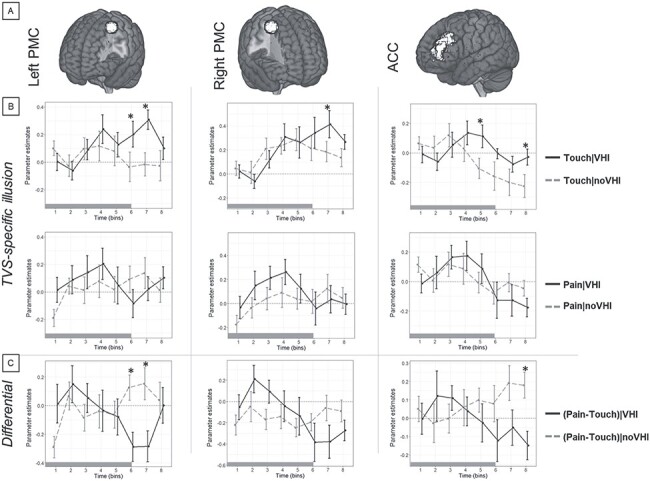
Transient activation, three-way interaction. (*A*) The “All Effects” analysis showed that the interaction between Illusion, TVS, and Time was significant in some of the preselected ROIs, namely the bilateral premotor (Left and Right PMC) and ACC. (*B*) The associated “TVS-specific illusion” analysis showed that, during vicarious touch, the activity of these regions was significantly higher after VHI than noVHI in late phases (time bins 5–8). These differences were not significant during vicarious pain. (*C*) The “Differential” analysis indicated that, in the left premotor and anterior cingulate cortices, the difference pain minus touch was higher after noVHI than VHI in late phases (time bins 6–8). Asterisks represent significant differences according to Tukey multiple comparisons tests over time bins. The bar under each plot represents the duration of videos showing vicarious touch/pain somatosensations.

Solving this issue, our All Effects analysis considered such timing-related differences within the transient activation in predefined ROIs and showed that the three-way interaction between Illusion, TVS, and Time was statistically significant in the left PMC, right PMC, and in ACC clusters (Fig. 5*A*, top row; Table 1). In addition, the associated TVS-specific illusion analysis showed that the transient brain activation during vicarious touch was higher after VHI than noVHI in: the left PMC at the sixth and seventh time bins; the right PMC at the seventh time bin; and the ACC at the fifth and eighth time bins from the onset of the vicarious somatosensation period (Fig. 5*B*, top row; Table 2). Conversely, during vicarious pain, the difference between the BOLD responses following the VHI and noVHI was not significant in any ROIs at any time bins (Fig. 5*B*, bottom row). Finally, the Differential analysis indicated that the transient brain activation for the difference pain minus touch was significantly higher after noVHI than VHI in the left PMC cluster at the sixth and seventh time bins, as well as in the ACC at the eighth time bin (Fig. 5*C*; Table 2).

**Table 1:** “All Effects” Analysis (Transient Activation). Significant effects (Effect) resulting from the three-way ANOVA between Illusion (VHI, noVHI), TVS (Touch, Pain), and Time (8bins), in the relevant region (Region), with the associated descriptive statistics

All Effects Analysis					
Effect	Region	eta^2^	*F*-value	DoF	*P*-value
Interaction (Illusion × TVS × Time)	Left PMC	0.23	4.10	7,98	0.01 (FDR)
Interaction (Illusion × TVS × Time)	Right PMC	0.15	2.54	7, 98	0.019 (unc)
Interaction (Illusion × TVS × Time)	ACC	0.14	2.35	7, 98	0.029 (unc)
Interaction (Illusion × Time)	Left ant Ins	0.13	2.07	7, 98	0.05 (unc)
Interaction (Illusion × Time)	preCun	0.17	2.78	7, 98	0.011 (unc)
Interaction (TVS × Time)	Left EBA	0.18	3.17	7, 98	0.04 (FDR)
Interaction (TVS × Time)	Left SMG	0.20	3.50	7, 98	0.04 (FDR)
Interaction (TVS × Time)	Right SMG	0.14	2.28	7, 98	0.03 (unc)
Interaction (TVS × Time)	Right PMC	0.14	2.24	7, 98	0.04 (unc)

**Table 2:** Further transient activation analyses. All the additional analyses following the All Effect analysis (Analysis). Only significant effects are reported (Effect), in the relevant region (Region), for the specific contrast between two conditions (Conditions) and their values (Mean ± SD), with the associated descriptive statistics

Analysis	Region	Time bin	Conditions	Mean ± SD	Cohen’s *d*	*t*-value	DoF	*P*-value
Differential	Left PMC	6th	VHI vs. noVHI	−0.29 ± 0.10 vs. 0.13 ± 0.09	1.13	2.71	67.8	0.0008
Differential	Left PMC	7th	VHI vs. noVHI	−0.28 ± 0.11 vs. 0.15 ± 0.11	1.04	2.87	67.8	0.006
Differential	ACC	8th	VHI vs. noVHI	−0.15 ± 0.08 vs. 0.18 ± 0.07	0.96	2.51	69.1	0.014
Illusion-related	Left ant Ins	5th	VHI vs. noVHI	0.26 ± 0.13 vs. −0.09 ± 0.08	0.41	2.01	55.5	0.05
TVS-related	Left EBA	5th	Touch vs. Pain	1.10 ± 0.13 vs. 0.86 ± 0.08	0.46	2.62	60.5	0.011
TVS-related	Left EBA	6th	Touch vs. Pain	1.00 ± 0.15 vs. 0.74 ± 0.15	0.45	2.89	60.5	0.005
TVS-related	Left EBA	7th	Touch vs. Pain	0.87 ± 0.13 vs. 0.67 ± 0.13	0.41	2.29	60.5	0.025
TVS-related	Left SMG	6th	Touch vs. Pain	0.42 ± 0.13 vs. 0.23 ± 0.13	0.43	2.71	71.5	0.008
TVS-related	Right SMG	6th	Touch vs. Pain	0.12 ± 0.08 vs. −0.06 ± 0.10	0.51	2.48	69.3	0.016
TVS-related	Right PMC	5th	Touch vs. Pain	0.27 ± 0.11 vs. 0.08 ± 0.10	0.51	2.48	69.3	0.016
TVS-related	Right PMC	6th	Touch vs. Pain	0.28 ± 0.11 vs. −0.01 ± 0.12	0.64	3.30	83.7	0.0014
TVS-related	Right PMC	7th	Touch vs. Pain	0.30 ± 0.10 vs. 0.08 ± 0.11	0.52	2.50	83.7	0.014
TVS-related	Right PMC	8th	Touch vs. Pain	0.20 ± 0.07 vs. 0.02 ± 0.08	0.60	2.06	83.7	0.04
TVS-specific illusion	Left PMC	6th	Touch|VHI vs. Touch|noVHI	0.20 ± 0.09 vs. −0.03 ± 0.11	0.61	2.50	105	0.014
TVS-specific illusion	Left PMC	7th	Touch|VHI vs. Touch|noVHI	0.31 ± 0.08 vs. −0.016 ± 0.07	1.08	3.42	105	0.0009
TVS-specific illusion	Right PMC	7th	Touch|VHI vs. Touch|noVHI	0.41 ± 0.11 vs. −0.018 ± 0.08	0.59	2.07	75.5	0.04
TVS-specific illusion	ACC	5th	Touch|VHI vs. Touch|noVHI	0.11 ± 0.08 vs. −0.10 ± 0.07	0.77	2.37	57.9	0.020
TVS-specific illusion	ACC	8th	Touch|VHI vs. Touch|noVHI	−0.02 ± 0.05 vs. −0.22 ± 0.08	0.76	2.14	57.9	0.04
TVS-specific illusion	Left ant Ins	3rd	Touch|VHI vs. Touch|noVHI	0.32 ± 0.09 vs. 0.09 ± 0.09	0.67	2.09	94.5	0.04
TVS-specific illusion	Left ant Ins	5th	Touch|VHI vs. Touch|noVHI	0.38 ± 0.11 vs. 0.05 ± 0.07	0.89	2.91	94.5	0.005
TVS-specific illusion	Left EBA	5th	Touch|VHI vs. Touch|noVHI	1.22 ± 0.14 vs. 0.97 ± 0.13	0.48	2.07	72.7	0.04
TVS-specific illusion	Left EBA	6th	Touch|VHI vs. Touch|noVHI	1.12 ± 0.15 vs. 0.88 ± 0.15	0.43	2.00	72.7	0.05
TVS-specific illusion	Left EBA	7th	Touch|VHI vs. Touch|noVHI	1.01 ± 0.13 vs. 0.74 ± 0.11	0.55	2.20	72.7	0.03

**Figure 6:**
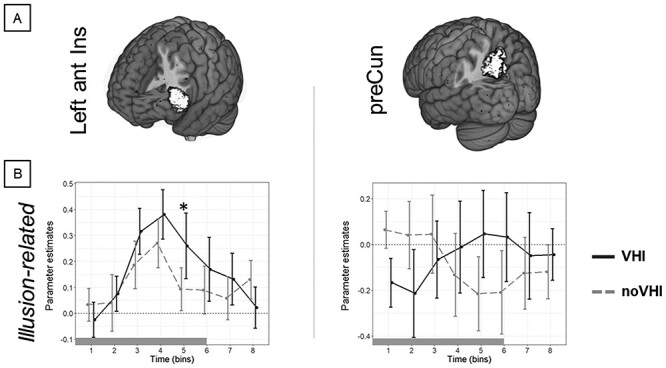
Interaction between Illusion and Time. The “All Effects” analysis showed that the interaction between Illusion (VHI, noVHI) and Time (8 bins) was significant in the left anterior insula (Left ant Ins) and precuneus (PreCun). The “Illusion-related” analysis showed that, for the insula at the 5th time bin, the neural activity was higher after VHI than noVHI. The asterisk represents statistically significant differences Tukey-corrected for multiple comparisons over time bins. The bar under each plot represents the duration of videos showing vicarious touch/pain somatosensations.

Furthermore, the All Effects analysis also showed significant two-way interactions between Illusion and Time in left ant Ins (Fig. 6*A*; Table 1). The associated Illusion-related analysis addressed the differences between VHI and noVHI separately for each Time bin and showed that transient brain activity in left ant Ins was higher after VHI than noVHI at the fifth time bin (Table 2; Fig. 6*B*). No Illusion-related differences were observed for the preCun at any Time bins. In addition, considering only vicarious touch (TVS-specific illusion analysis), transient brain activity was higher for VHI compared with noVHI in left ant Ins at the third and fifth time bin (Supplementary Fig. 3; Table 2). The difference between VHI and noVHI during vicarious pain was not significant in any of these ROIs (left ant Ins and preCun) for any of the time bins.

**Figure 7:**
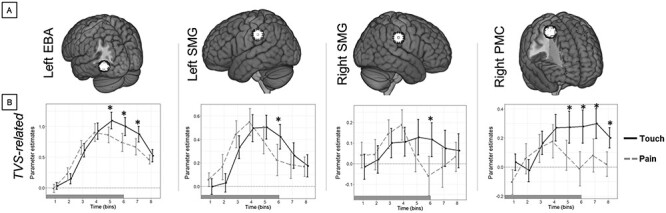
Interaction between TVS and Time. The “All Effects” analysis showed that the interaction between TVS [touch (black line), pain (gray line)] and Time (8 bins) was significant in the Left EBA, bilateral SMG, and right PMC. The “TVS-related” analysis showed that in all these regions, the neural activation following vicarious touch was higher than that for vicarious pain, specifically in late phases (time bins 5–8). Asterisks represent significant differences Tukey-corrected for multiple comparisons over time bins. The bar under each plot represents the duration of videos showing vicarious touch/pain somatosensations.

Finally, the All Effects analysis also showed significant two-way interactions between TVS and Time in left EBA, left SMG, right SMG, and right PMC (Fig. 7*A*; Table 1). Post-hoc comparisons (TVS-related analysis) indicated that transient brain activity during vicarious touch was higher than vicarious pain in: left EBA at the fifth, sixth, and seventh time bins; left SMG at the sixth time bin; right SMG at the sixth time bin; and in right PMC at the fifth, sixth, seventh, and eighth time bins (Table 2; Fig. 7*B*). Furthermore, with the TVS-specific illusion analysis, we observed that, during vicarious touch, transient brain activity was higher for VHI compared with noVHI in left EBA at the fifth, sixth, and seventh time bins, as well as in the right PMC at the seventh bin (Supplementary Fig. 4; Table 2). The difference between VHI and noVHI during vicarious pain was not significant in any of these ROIs (left EBA, left and right SMG, and right PMC) at any time bins.

## Discussion

At the theoretical level, the present study investigated the influence of altered body ownership on the neural underpinnings of vicarious somatosensation, with a focus on the influence of different temporal profiles of brain activation for different types of vicarious somatosensations. Accordingly, at the methodological level, instead of using the same HRF to model both vicarious touch and vicarious pain, we used a time-course analysis that was crucial for temporally characterizing the brain activation differences. This approach provided a time-sensitive view on how low-level sensory input (visuo-tactile stimulation) would be able to affect higher level cognitive processing (body ownership) which, in turn, would be associated with relatively late-epoch inhibition of premotor and cingular activity related to vicarious pain (not vicarious touch), only after the visuo-tactile-dependent alteration of body ownership.

The use of a robot excluded any potential bias due to the presence of an experimenter in the MR room via a human-action-free automated procedure (Limanowski et al. 2014; Bekrater-Bodmann et al. 2014). The experiment was further controlled by applying the same loadings of visual and tactile stroking stimulations also in the noVHI (asynchronous) condition, which is known to decrease or eliminate the VHI (Perez-Marcos et al. 2012; Pyasik et al. 2020). This procedure allowed to rule out any potentially unintended factors which might have biased the findings, including visual habituation or passive stroking.

### Altered Body Ownership Affects Transient Brain Activity during Specific Vicarious Somatosensations

#### Temporo-Parietal Junction

At a first glance, the results based on the sustained activity approach might suggest that during the experience of vicarious pain, the left TPJ was more active when the virtual hand was illusorily incorporated into participants’ body ownership (VHI condition), with respect to when no illusion is expected (noVHI condition; Fig. 4). Showing a sensed left lateralization in association with the visuo-tactile stimulation of the right (virtual and participant’s) hand and vicarious somatosensation, this finding would be in line with previous evidence that TPJ has an established role in visuo-tactile multisensory integration (Matsuhashi et al. 2004; Arzy et al. 2006; Grivaz et al. 2017), body representation/ownership (Tsakiris et al. 2008; Ionta et al. 2011; Convento et al. 2018; Zeugin et al. 2020), and vicarious somatosensation (Silani et al. 2013; Benuzzi et al. 2018; Flasbeck et al. 2019; Ionta et al. 2020; Tholen et al. 2020). However, this interpretation would become less straightforward when considering that the activation during vicarious touch was not independent from body ownership, but rather it was significantly lower after VHI than noVHI. We propose that the possibly counterintuitive findings based on the Sustained Activation Analysis might be the result of using the same HRF in BOLD signal analysis for two different events (vicarious touch vs. vicarious pain).

#### Premotor and ACC

As suggested by the present sustained activation results, the analysis of fMRI data using a block-related HRF model might be less sensitive to event-specific temporal changes in brain activity. To address this issue, at least at the level of the temporal resolution of the functional acquisition, we adopted the FIR approach by dividing the block of vicarious somatosensation into shorter epochs (time bins) and introduced the new factor Time in the following analyses. In this way, the All Effects analysis showed the significant interaction between Illusion, TVS, and Time in the activity of bilateral PMC and ACC (Fig. 5). The Time-specific analysis further specified that only in relatively late epochs of vicarious somatosensation, the activity in these regions during vicarious touch was higher after the alteration of body ownership (VHI) with respect no alteration (noVHI) (Table 2). Conversely, in these regions, the neural activity during vicarious pain was not significantly different following VHI or noVHI at any epochs. The “Differential” analysis showed that such effect was driven by the fact that for left PMC and ACC in late epochs of vicarious somatosensation, the differential activity for vicarious pain minus vicarious touch was negative (lower activation for vicarious pain) after VHI, while it was positive (higher activation for vicarious pain) after noVHI. This finding established that, particularly for the left premotor and anterior cingulate cortices, such effect was driven by the lower BOLD signal after the embodiment of the virtual hand (VHI) associated with vicarious pain with respect to vicarious touch.

Activations in bilateral PMC were stronger when the vicarious touch followed VHI than noVHI in late epochs of vicarious somatosensation. This suggests that a change in body ownership influenced the brain activity associated with vicarious touch, but specifically at late stages. Previous work showed that premotor activations are associated with both altered body ownership (Ehrsson et al. 2004; Ehrsson et al. 2005; Bekrater-Bodmann et al. 2014) and vicarious somatosensations (Keysers et al. 2004; Costantini et al. 2008; Ochsner et al. 2008; Ushida et al. 2008; Lamm et al. 2011). In this vein, our results provide evidence supporting what previously could be only postulated as a neural basis of the link between body ownership and vicarious somatosensation at the level of the PMC. Considering that both the virtual hand’s incorporation and vicarious somatosensation imply a certain degree of mental spatial transformation of one’s own hand representation in space (illusory mislocalization), it is not surprising that the PMC is specifically recruited, given its crucial role in the recalibration of the spatial coordinate system for the hand position (Tsakiris 2010), a widely accepted consequence of the VHI (Slater et al. 2008; Perez-Marcos et al. 2012; Patterson et al. 2017; Lim et al. 2020; Pyasik et al. 2020). Here, we propose that such a proprioceptive recalibration might be the origin of the increased neural activity associated with the vicarious somatosensation of touch, especially at relatively late stages (fourth to eighth time bins). The stimulation related to the vicarious somatosensation (virtual hand being touched) displayed the touching object in a space far from the participants’ hand located about 2 m in front of the participants’ eyes instead of next to their leg. Due to the proprioceptive recalibration brought by the VHI, we propose that participants might have had the (conscious or unconscious) illusion that their right hand was located closer to or overlapping with the virtual hand. In this vein, it might be the case that the presence of this illusory mislocalization (VHI) triggered an augmented neural activity for vicarious touch, which might have kept the activity of the PMC stronger for a longer time with respect to the absence of illusory mislocalization (noVHI). This interpretation would explain also why the neural influence of altered body ownership (through synchronous visuo-tactile stimulation) on vicarious touch occurred only in late stages. Given that visual and tactile stimulation occurred in reasonably coherent spatial locations, the activation of the PMC in the first stages (first to fourth time bin) might be the result of the mere visuo-tactile stimulation regardless of visuo-tactile/proprioceptive synchrony (Macaluso and Maravita 2010; Brozzoli et al. 2012; Limanowski and Blankenburg 2016; Convento et al. 2018).

In line with the activation patterns of the PMC, we also found that activity in ACC, especially in late epochs, was higher when the vicarious touch was preceded by VHI instead of noVHI. Such a body-ownership-related difference was not significant for the brain activity associated with vicarious pain. Like the PMC, neural activity in ACC is also sensitive to both altered body ownership (Lloyd et al. 2006; Ehrsson et al. 2007) and vicarious somatosensations (Singer et al. 2004; Gu et al. 2012; Keum and Shin 2019; Ionta et al. 2020). However, its role is more related to processing the affective aspects of vicarious (Lamm et al. 2011; Bzdok et al. 2012; van der Heiden et al. 2013; Tholen et al. 2020) and illusory (Craig et al. 1996) somatosensations. We propose that ACC activity was higher in the late stage of vicarious touch because its activation patterns reflected those of the PMC. Thus, the activation pattern of ACC would reflect the neural encoding of the affective aspects of vicarious touch, which would be enhanced following the VHI. Since both ACC and premotor activations were higher in late stages of vicarious touch, such enhancement might occur after the proprioceptive recalibration but in parallel with the reactivity to the sensorimotor aspects of vicarious touch encoded by the PMC. As for the PMC, the possible illusory mislocalization of the participants’ hand toward the virtual hand, induced by the previous synchronous visuo-tactile stimulation, might have produced an augmented sensitivity to affective components of vicarious touch, keeping the activity of ACC higher for a longer time, with respect to the period of vicarious touch following the asynchronous visuo-tactile stimulation.

Why was not vicarious pain associated with different VHI-dependent patterns of activation? One possible explanation is that vicarious pain is semantically stronger than vicarious touch, implying higher body ownership, and therefore, the neural activity associated with it would be less sensitive to illusory changes in body ownership elicited by the VHI. This idea is in line with the observation that vicarious pain is perceived as more intense than other vicarious somatosensations (Holle, Banissy, et al. 2011; Fusaro et al. 2016; Fusaro et al. 2019). Accordingly, in the present study, it might be that the changes in illusory body ownership were not strong enough to affect the neural response to vicarious pain in the PMC and ACC. Perhaps, an experimental protocol able to elicit stronger changes in body ownership might also modulate the neural response to vicarious pain.

### The Dynamic Influence of Altered Body Ownership on Vicarious Somatosensation

#### Insula

The “All Effects” analysis also showed that during vicarious somatosensation, regardless of its type, the dynamic activity in the left anterior insula and precuneus was different after VHI compared with noVHI (Fig. 6, Table 2). The associated “Time-specific” analysis showed that, for left insula, dynamic activity in a late stage from the onset of vicarious somatosensation was higher after VHI than noVHI (Fig. 5), a result that was driven by vicarious touch (Supplementary Fig. 3). The insular cortex has been repeatedly associated with processing of the somatosensory aspects of altered body ownership, including interoception awareness (Ehrsson et al. 2007), subjective experience (Tsakiris 2010), and vicarious sensations (Singer and Lamm 2009). Similarly, also the precuneus is involved in internally oriented processing, imagined sensation from a first-person perspective (Lamm et al. 2007), and it is part of a vicariousness-related brain network (Tholen et al. 2020). On this basis, we propose that a change in body ownership driven by synchronous visuo-tactile stimulation might have induced stronger self-referred vicarious feelings and sensations for the virtual hand which, in turn, were reflected in higher dynamic activation of the insula and precuneus after VHI compared with noVHI. Showing that the neural effects of altered body ownership do not stop with the end of the visuo-tactile stimulation, but rather they linger and can interfere with the neural activity of a supposedly unrelated task (vicarious somatosensation), these findings support that changes in body ownership affect the neural underpinnings of vicarious somatosensation.

### The Role of Different Types of Vicarious Somatosensations

#### Extrastriate, Supramarginal, and Premotor Activity

The “All Effects” analysis finally showed that the left EBA, bilateral SMG, and right PMC were more active during vicarious touch than vicarious pain, regardless of the alteration or not of body ownership (VHI, noHVI). The “Time-specific” analysis further indicated that, specifically in late epochs, the brain activity in all these regions was higher during vicarious touch than vicarious pain (Fig. 6). In addition to the interpretation of the premotor activation discussed above, an increased activity in EBA has been related to illusory body ownership (Limanowski et al. 2014), self-identification with a body (Ionta et al. 2011), vision of a body or body parts (Downing et al. 2001), mental imagery of embodied self-location (Arzy et al. 2006) and, importantly, with the integration of body representation and somatosensory information (Costantini et al. 2011). On this basis, we propose that the weaker activity of EBA during vicarious pain might be the consequence of a stronger cognitive resistance against, or avoidance from (Harlé et al. 2021), the sensory information related to the virtual hand during vicarious pain than vicarious touch, suggesting the activation of more autonomic aspects of body representation during vicarious pain with respect to vicarious touch. On a similar account, also the activity of bilateral SMG for vicarious pain started to decrease earlier than the one for vicarious touch. As part of TPJ, SMG is a key region for a vicariousness-related network (Tholen et al. 2020). It is linked to the integration of multisensory information (Gentile et al. 2011), is particularly important for processing the somatosensory aspects of vicarious somatosensation (Lamm et al. 2011; Bzdok et al. 2012; Ionta et al. 2020; Tholen et al. 2020), and is more active during first-person than third-person experiences (van der Heiden et al. 2013). In the present study, the visual presentation for vicarious pain (hand being pricked by a syringe needle) and vicarious touch (hand being touched by the bottom of the syringe) could have components of empathy (the capacity one has to feel what another is experiencing) and a component of perspective taking (perceiving a situation from an external point of view). It was reported that brain activation from both receiving and observing painful stimulations in others overlaps with some regions of the pain matrix, including the SMG (Costantini et al. 2008; Ferraro et al. 2012; Castillo et al. 2018). In our study, we observed that the SMG responds to vicarious pain differently than to vicarious touch: viewing the virtual hand receiving painful stimulation, regardless of alteration of body ownership, leads to the reduction of neural activity in SMG. Conversely, SMG sustains activity for longer when the vicarious experience relates to touch. Similar results are observed in the left EBA and right PMC. Altogether, we propose that the earlier decrease of activity in EBA, SMG, and PMC during vicarious pain with respect to vicarious touch may be the result of an automatic avoidance reflex (Johnston et al. 2015) associated with the vicarious pain, which would implicitly push participants to take a larger psychological distance from the virtual hand in pain than in touch, aiming to decrease the valence of (painful) somatosensory aspects of vicarious somatosensations. These defensive mechanisms might be due to an effective strategy supporting self-generated, autonomic avoidance of aversive nociceptive visual stimulation, even though vicarious.

### Psychological Constructs

The RHI Qs results suggested that the present experimental protocol was able to induce alterations of body ownership based on the VHI, a variant of the RHI: a well-known procedure to induce illusory body ownership (incorporation of the rubber hand into one’s own body representation; Armel and Ramachandran 2003) and bodily reallocation (sensation that tactile input is perceived from a rubber hand; Kammers et al. 2009). Thus, rather than a permanent construct, body representation seems to be flexible and alterable as a function of sensory visuo-tactile input. In addition, the IRI scores indicated that participants were sensitive enough to emotionally react to vicarious somatosensations, with relatively higher scores in the Empathic Concern and Personal Distress subscales of the IRI, with respect to Perspective Taking and Fantasy Scale. Empathic Concern and Personal Distress refer to affective reactivity (FeldmanHall et al. 2015), while Perspective Taking and Fantasy Scale refer to cognitive reactivity (Shamay-Tsoory et al. 2009). It is therefore likely that our participants had a larger resonance for cognitive aspects of vicarious somatosensation, in line with the idea that low-level sensory conflicts induce changes in cognitive aspects of body representation.

Altogether, the present study suggests that the induction of illusory body ownership (VHI) modulated the neural correlates of vicarious somatosensations. The fact that this finding was more significant during vicarious touch than vicarious pain might suggest that some levels of embodiment are already present when participants observe a painful stimulation. This would also be in line with the idea that vicarious pain is somehow more salient than vicarious touch. In this way, a hand-related visuo-tactile coherence may be able to substantially alter body ownership during vicarious somatosensation of touch. Therefore, since the vicarious somatosensation of pain per se may induce effects similar to an incorporation of the virtual hand, a limited capacity for additional shift of body ownership would remain available for the VHI.

These findings provide new empirical evidence that the impact of sensory processing is not limited to body ownership but extends also to vicarious experience. In the present study, the integration of vision (virtual hand stroking; “visual stroking”) and touch (robotic stroking of the participant’s hand; “tactile stroking”), the initial part of each experimental cycle was considered a relatively “lower level” perceptual process. On the other hand, the “higher level” cognitive processing concerned the other body-related psychological aspects (vicarious somatosensation) affected by the previous visuo-tactile stimulation. While it has been repeatedly demonstrated that visuo-tactile stimulation affects body ownership, the “step forward” of the present study is the finding that (low-level) visuo-tactile-dependent alterations are not limited to body ownership but, rather, encompass also other high-level body-related psychological domains, such as vicarious somatosensation. Some previous behavioral observations suggested the link between body ownership and vicarious somatosensation (Botan et al. 2018), vicarious action (Cioffi et al. 2020), and vicarious emotions (Asai et al. 2011). The present study confirms previous behavioral findings and further adds new knowledge about the neural correlates of body-related, low/high, sensory/cognitive interactions. In particular, the activation patterns of the PMC and ACC suggest that visuo-tactile stimulation affected the neural resonance mechanisms associated with vicariousness. While PMC is widely accepted as one of the key brain regions activated by altered body ownership due to multisensory conflicts (Ehrsson et al. 2004), ACC has been linked to the processing of emotional (not sensory) aspects of vicarious somatosensations (Singer et al. 2004). On this basis, we propose that in the present study, the visuo-tactile conflict affected the emotional aspects of vicarious somatosensation (ACC) mediated through the motor resonance mechanisms (PMC) shared among vicariousness and body ownership.

### Temporal Dynamics

Substantial differences related to the interaction between altered body ownership and vicarious somatosensations were observed only in relatively late epochs after the onset of the vicarious phase (12–16 s, Fig. 5). As shown by the post-hoc analysis of the three-way interactions, this might be a product of reduced activity following stimulus-related activation for vicarious pain in addition to both delayed peak and sustained activity for vicarious touch, after the induction of altered body ownership (VHI). This was a common pattern observed across other regions in our study (see, e.g., left anterior insula and left SMG in Figs 6 and 7, respectively). In fact, van der Heiden (2013) observed that self-oriented perspective enhances activation in the left SMG compared with adopting a perspective related to the other, which is consistent with our assumption that enhanced embodiment led to a more self-centered perspective. The same study noted that self-orienting leads to higher early peaks related to pain experience, which reflects an intuitively more autonomic process because of enhanced body ownership. Together with our findings, this evidence helps elucidate the neural correlates of how embodiment changes one’s experience. Altered body representation after RHI leads to somatosensory effects, such as decreased skin temperature (Moseley et al. 2008), reduced perception of touch on the real hand (Kilteni and Ehrsson 2017), and lower diminished perception of received pain on the real hand after illusion (Fang et al. 2019). These effects are in line with our results for brain activity, in which the neural effects also indicate altered self-oriented perspective after the induced embodiment illusion (Ehrsson et al. 2007; Gentile et al. 2015).

### Sustained versus Transient Approach

Using a block design, we observed that TPJ exhibits differences due to the same effects in sustained activity (Fig. 4*A*,*B*). However, as it is suggested by Figure 4*C*, this difference is better understood when we consider neural activity over time, rather than estimating activation through a model that implies sustained activity in the block. In our study, the analysis of transient differences in the BOLD signal across conditions was crucial to determine that enhanced embodiment may alter the way participants experience a vicarious stimulus. Our results corroborate the idea that temporal characteristics of brain activity—not only its magnitude—are an important approach to distinguish different conditions (Wang et al. 2021). As a corollary of our study, we argue that an analysis that relies exclusively on the assumption of predefined response models fixed across condition blocks might eventually be suboptimal for capturing neural responses in conditions that evoke different activity, ultimately leading to erroneous inferences on brain correlates (Fig. 4). In fact, HRF variations may affect statistical analyses, misestimate magnitudes, and lead to false negatives (Handwerker et al. 2004; Wang et al. 2021). Variability in HRF exists across participants, sessions, and brain regions (Aguirre et al. 1998; Handwerker et al. 2004), as well as for different tactile stimuli (Wang et al. 2021). The absence of transient signal analysis might explain why some studies failed to replicate effects related to the RHI in the PMC (Limanowski et al. 2014; Apps et al. 2015). Further studies are necessary to examine whether and to which extent this observation arises in similar designs or cognitive factors.

As seen here, FIR time-courses can capture transient changes in predefined brain regions in terms of the shape of responses to different conditions. If one aims to map the brain when the HRF is likely to change across conditions, one alternative could be using part of the dataset to first estimate the HRF and then applying it to the remaining portion of the dataset to analyze whole-brain activity. Another consideration is that relatively long times of stimulation are a common feature in experiments involving empathy or perspective taking (Singer et al. 2004; Lamm et al. 2007; van der Heiden et al. 2013). Similar to other experiments (van der Heiden et al. 2013), our study used a relatively long interval of vicarious stimulation (12 s) in order to capture early bottom-up, automatic responses, as well as top-down, late regulatory responses related to empathy or perspective taking.

### Limitations

#### Sample Size

One limitation of our study might be that the sample size might be considered modest and therefore limit our conclusions. However, to evaluate the statistical power of a study, it is important to consider especially the effect size of the significant effects. For this reason, we computed Cohen’s *d* or eta squared for each significant effect, which fell within the range of commonly accepted effect sizes. A larger sample size would likely elucidate some results that were here only evidenced as a trend. For example, although not statistically significant, it might be noted that in the early epochs of vicarious somatosensation, there was a trend for higher magnitude in brain activity for the vicarious pain than vicarious touch (Fig. 5 and Supplementary Figs 3 and 4). Although we cannot infer that our experiment induced an activation switch for the vicarious somatosensation (i.e., vicarious pain for early epochs and vicarious touch for late epochs), it would remain in line with our main conclusion that the weaker activations for vicarious pain in late epochs might reflect a stronger avoidance reflex with respect to vicarious touch, particularly in late epochs of vicarious somatosensation.

#### Psychological States (RHI Qs)

Although the obtained results seem encouraging, it is possible that the RHI Qs results should be regarded with caution, based on two main reasons. First, due to technical limitations, our participants completed the RHI Qs at the end of the experiment, instead of right after each block of visuo-tactile stimulation. Such a temporal gap between the experience of the illusion and the participants’ answers might constitute a confounding factor. Second, we used only one measure of subjective aspects of the illusion (questionnaires). Previous work suggests that such a “unilateral” approach may be not optimal, because findings based on only one measure do not allow to exclude false positives due to the absence of another (control) measure and, indeed, different measures of the RHI do not necessarily correlate to each other. For example, proprioceptive drift does not correlate with mental rotation (Ionta et al. 2013) and questionnaire scores do not necessarily correlate with proprioceptive drift (Rohde et al. 2011; Holle, McLatchie, et al. 2011), skin conductance (Tieri et al. 2015), pain perception (Mohan et al. 2012), body awareness (David et al. 2014), and movement kinematics (Kammers et al. 2009).

## Conclusions

At the theoretical level, the present study shows that altered body ownership differentially affects the neural underpinnings of vicarious pain versus touch, especially in relatively late epochs (time bins) of the vicarious experience. The enhanced embodiment of the virtual hand was associated with inhibited cortical response for vicarious experience of pain compared with vicarious experience of touch. Focusing on how the transient brain signals evolved over time during vicarious somatosensations as a function of visuo-tactile stimulation allowed a better understanding of the dynamicity of the neural influence of body ownership on vicarious somatosensation. This approach provided evidence that the “low-level” multisensory conflict affects both “high-level” body ownership and vicarious experience, being the PMC the possible neural bridge (or shared neural underpinning) between these two aspects of body representation.

At the methodological level, the combination of MR-compatible robotics and virtual reality ensured that all participants systematically received the same, well-controlled, and reproducible multisensory input. Building on this setup, the present study applied a time-sensitive approach to the investigation of sensory-cognitive interactions. Although solidly established in fMRI procedures, “HRF-fixed” data analysis approaches (canonical HRF) that apply the same HRF to all experimental conditions might be not sensitive enough to capture neurally dynamic relationship between body ownership and vicariousness, due to the hemodynamic differences between conditions. Conversely, in the context of the present study, our “HRF-flexible” approach (FIR model) took into account flexible hemodynamic responses across different experimental conditions, allowing us to observe that different sensory-cognitive interactions between body ownership and somatosensory vicariousness are reflected in different temporal patterns of brain activity.

Altogether, showing that altered body ownership modifies the hemodynamic response associated with vicarious experiences, we propose that the present study may 1) help to better understand how multisensory integration contributes to build a coherent sense of body representation and 2) provide the basis for advances in clinical applications in reaction to disorders affecting body perceptions and multisensory integration (e.g., autoscopy, somatoparaphrenia, and dissociative disorders).

## Supplementary Material

RobotEmpathy_Final_32_SuppMat_bhab210

## Abbreviations

RHI (rubber hand illusion), VHI (virtual hand illusion), MR (magnetic resonance), fMRI (functional magnetic resonance imaging), GLM (general linear model), FIR (finite impulse response), BOLD (blood-oxygen-level-dependent), TVS (type of vicarious somatosensation), MNI (Montreal neurological institute), TR (repetition time), TE (echo time), FDR (false discovery rate), PMC (premotor cortex), ACC (anterior cingulate cortex), ant Ins (anterior insula), preCun (precuneus), TPJ (temporo-parietal junction), EBA (extrastriate body area), Thal (thalamus), S1 (primary sensory cortex), S2 (secondary sensory cortex), SMG (supramarginal gyrus), ANOVA (analysis of variance), HRF (hemodynamic response function), IRI (Interpersonal Reactivity Index).

## Notes

We thank the Translational Neuromodeling Unit (ETH Zurich) for advice on the data analysis. *Conflict of Interest:* All authors declare no conflict of interest.
