# Supplementary material for: Illusory Body Ownership Affects the Cortical Response to Vicarious Somatosensation
Source: Cereb Cortex. 2021 Jul 8;32(2):312–28. doi: 10.1093/cercor/bhab210 (PMC8754387; doi:10.1093/cercor/bhab210)
Supplement: RobotEmpathy_Final_32_SuppMat_bhab210 [file robotempathy_final_32_suppmat_bhab210.docx]

**SUPPLEMENTARY MATERIAL for:**

**"Illusory body ownership affects the cortical response to vicarious somatosensation".**

**Table S1 - Regions of interest**


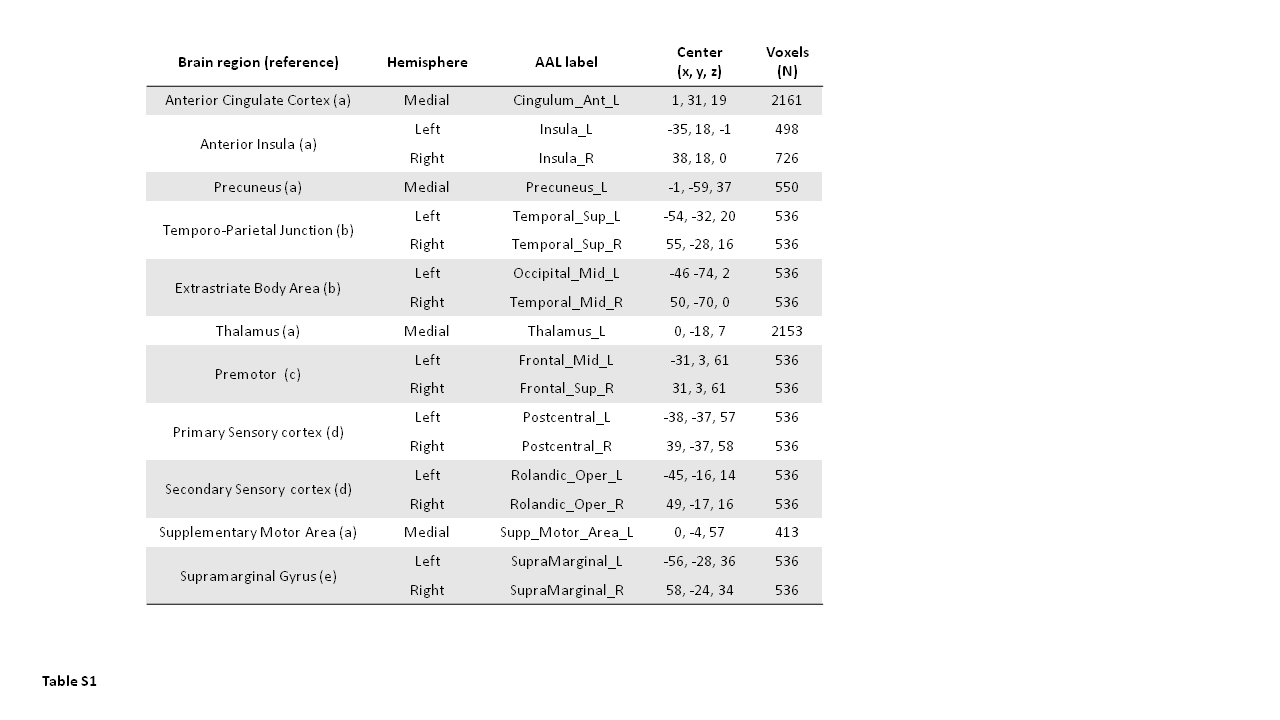


**Table S1 - Regions of Interest (ROIs).** ROIs were identified on the basis of previous topic-related publications: (a) Yarkoni, et al., 2011, Nature Methods; (b) Ionta, et al., 2011, Neuron; (c) Mayka, et al., 2006, NeuroImage ; (d) Bingel, et al., 2004, NeuroImage; (e) Flasbeck, et al., 2019, Front Psychiatry. Each ROI (Brain region) was localized either in the left/right hemisphere or could comprise medial structures (Hemisphere), was automatically labeled according to the Automated Anatomical Labeling (AAL) atlas, was centered on the reported coordinates (Center), and comprised the indicated number of voxels (Voxel).

**Figure S1**


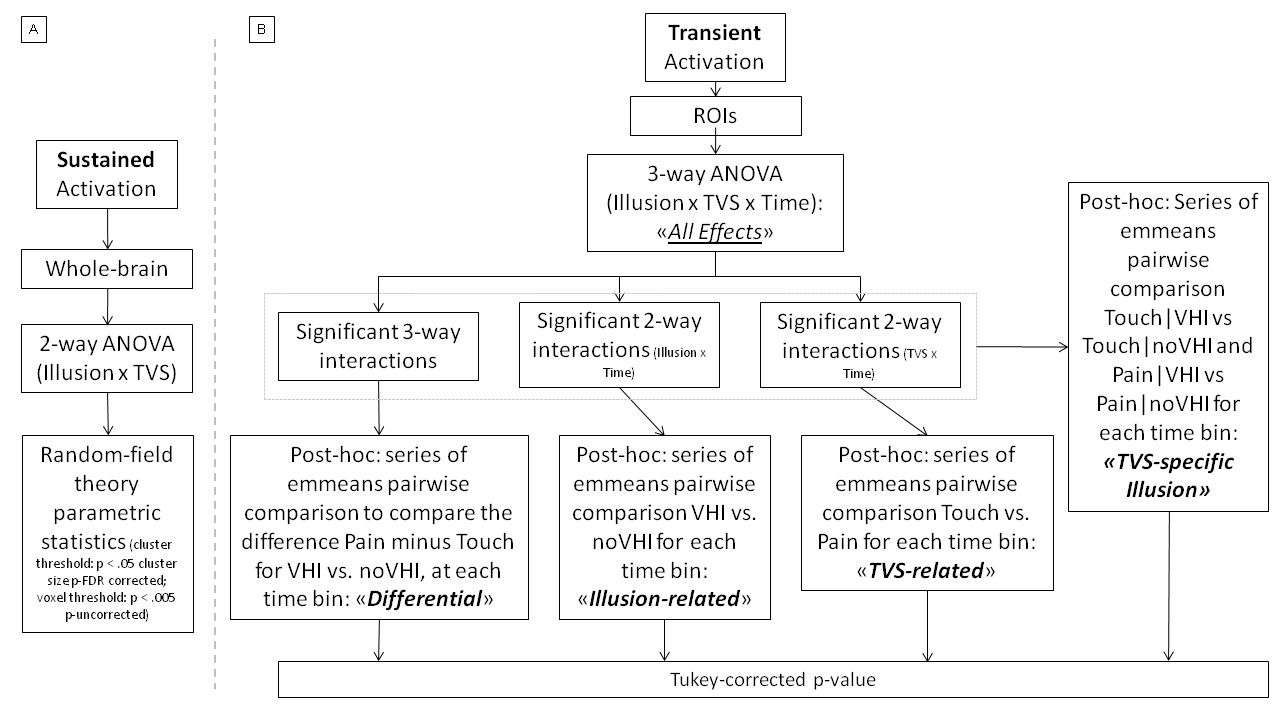


**Figure S1 - fMRI analysis approach.** **A) Sustained** activation was analyzed according to a whole-brain approach, using the same hemodynamic model for all conditions and running a 2-way ANOVA between Illusion and TVS (random field theory - cluster threshold: p < .05 cluster size p-FDR corrected; voxel threshold: unc. p < .005). **B) Transient** activation was analyzed in a set of predefined ROIs, using different time-sensitive models (finite impulse response basis functions) for each condition to obtain peristimulus time-courses. This resulted in the 3-way repeated-measures ANOVA between Illusion, TVS, and Time. Further post-hoc comparisons were run in order to test specific effects (*Differential, Illusion-related, TVS-related, TVS-specific*). Other abbreviations: TVS (type of vicarious somatosensation); FDR (false discovery rate), ROIs (regions of interest).

**Figure S2**


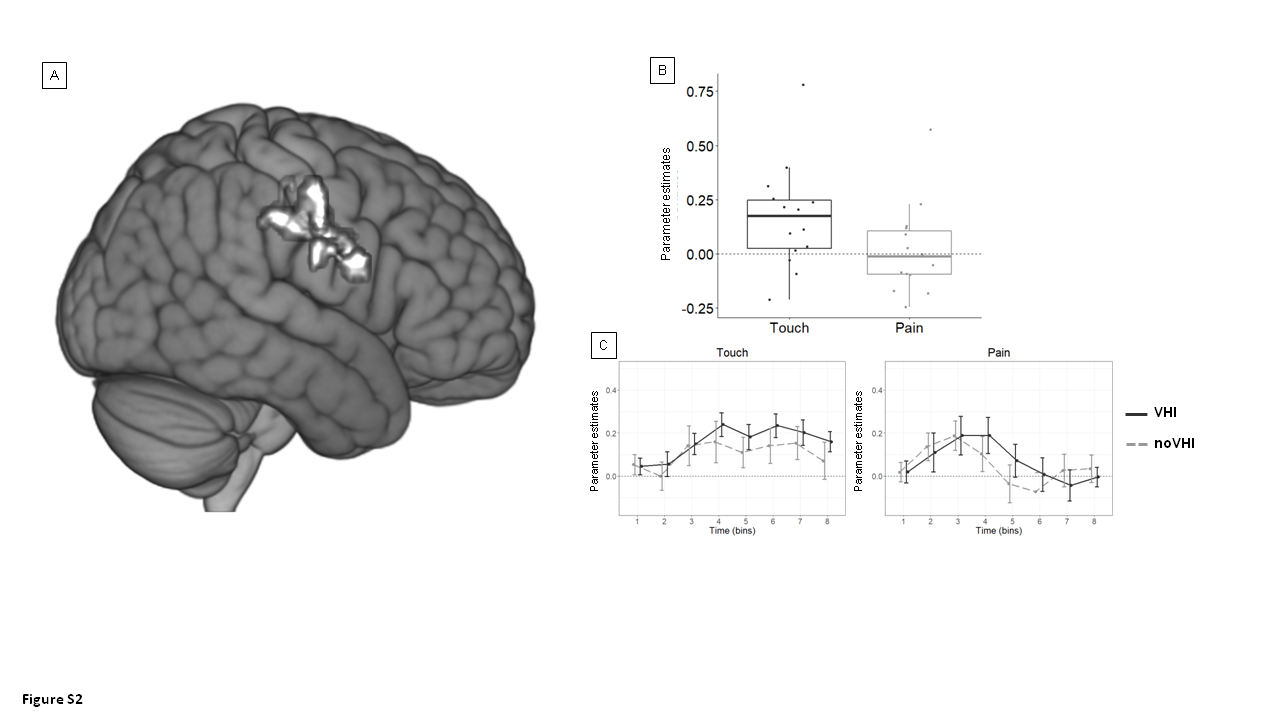


**Figure S2 - Sustained activation, main effect of TVS**. **A)** The cluster comprising portions of the right pre/post-central and inferior frontal gyri was found in the whole-brain analysis as the only one in which brain activity was significantly different between vicarious touch (higher) and vicarious pain (lower). **B)** The sustained activity in this cluster was higher during vicarious touch compared to vicarious pain. **C)** Temporal profiles extracted from the detected cluster are shown for each condition separately. Here, we observe decreased activity in late epochs for vicarious pain compared to vicarious touch. Abbreviations: TVS (type of vicarious somatosensation).

**Figure S3**


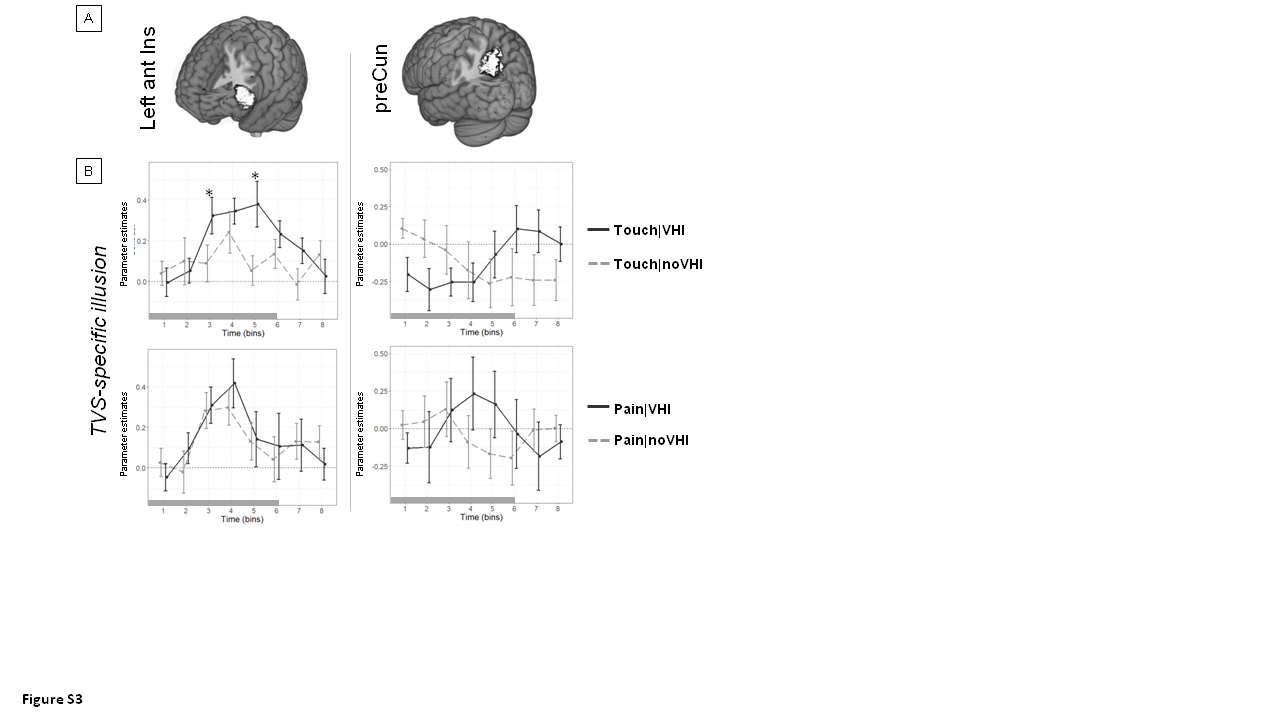


**Figure S3 – TVS-specific illusion time-courses for ROIs with significant Illusion by Time interaction.** Time-courses for each condition are plotted separately for ROIs with significant interaction Illusion x Time [left anterior insula (left ant Ins) and precuneus (preCun)]. During vicarious touch, the insular activation was higher after VHI than noVHI at the 3rd and 5th time bins. Asterisks represent significant differences according to Tukey multiple comparisons tests over time bins. The gray bar under each plot represents the duration of videos showing vicarious touch/pain somatosensations. Other abbreviations: TVS (type of vicarious somatosensation); ROIs (regions of interest).

**Figure S4**


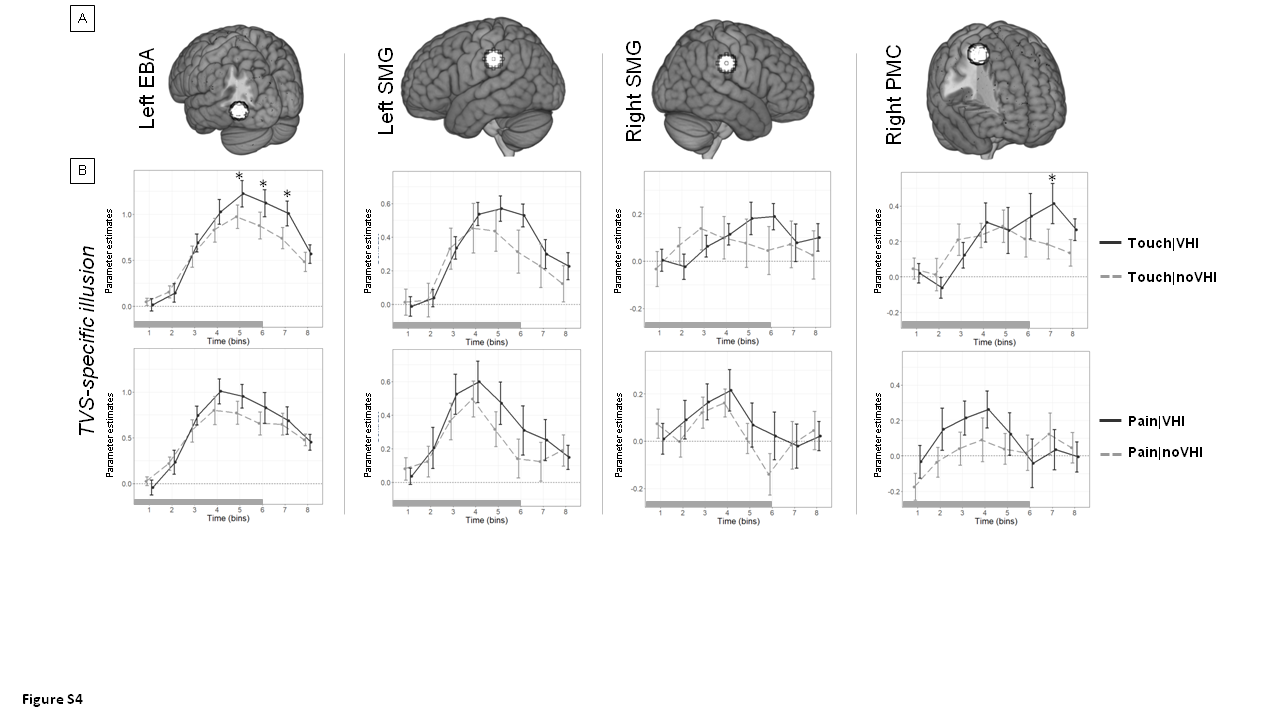


**Figure S4 - TVS-specific illusion time-courses for ROIs with significant TVS by Time interaction.** Specifically for the left EBA and right PMC, brain activity was higher after VHI than noVHI in late epochs of vicarious touch. Asterisks represent significant differences according to Tukey multiple comparisons tests over time bins. The gray bar under each plot represents the duration of videos showing vicarious touch/pain somatosensations. Abbreviations: EBA (extrastriate body area); PMC (premotor cortex); TVS (type of vicarious somatosensation); VHI (virtual hand illusion).
